# Myocardial Perfusion SPECT Imaging Radiomic Features and Machine Learning Algorithms for Cardiac Contractile Pattern Recognition

**DOI:** 10.1007/s10278-022-00705-9

**Published:** 2022-11-14

**Authors:** Maziar Sabouri, Ghasem Hajianfar, Zahra Hosseini, Mehdi Amini, Mobin Mohebi, Tahereh Ghaedian, Shabnam Madadi, Fereydoon Rastgou, Mehrdad Oveisi, Ahmad Bitarafan Rajabi, Isaac Shiri, Habib Zaidi

**Affiliations:** 1 Department of Medical Physics, School of Medicine, Iran University of Medical Science, Tehran, Iran; 2 Rajaie Cardiovascular Medical and Research Center, Iran University of Medical Science, Tehran, Iran; 3 Division of Nuclear Medicine and Molecular Imaging, Geneva University Hospital, CH-1211 Geneva 4, Switzerland; 4 Department of Biomedical Engineering, Tarbiat Modares University, Tehran, Iran; 5 Nuclear Medicine and Molecular Imaging Research Center, School of Medicine, Namazi Teaching Hospital, Shiraz University of Medical Sciences, Shiraz, Iran; 6 Comprehensive Cancer Centre, School of Cancer & Pharmaceutical Sciences, Faculty of Life Sciences & Medicine, King’s College London, London, UK; 7 Echocardiography Research Center, Rajaie Cardiovascular Medical and Research Center, Iran University of Medical Sciences, Tehran, Iran; 8 Cardiovascular Interventional Research Center, Rajaie Cardiovascular Medical and Research Center, Iran University of Medical Sciences, Tehran, Iran; 9 Geneva University Neurocenter, Geneva University, Geneva, Switzerland; 10 Department of Nuclear Medicine and Molecular Imaging, University of Groningen, University Medical Center Groningen, Groningen, Netherlands; 11 Department of Nuclear Medicine, University of Southern Denmark, Odense, Denmark; 12 Department of Computer Science, University of British Columbia, Vancouver BC, Canada

**Keywords:** Machine learning, CRT, GSPECT MPI, Radiomics, Quantitative features

## Abstract

A U-shaped contraction pattern was shown to be associated with a better Cardiac resynchronization therapy (CRT) response. The main goal of this study is to automatically recognize left ventricular contractile patterns using machine learning algorithms trained on conventional quantitative features (ConQuaFea) and radiomic features extracted from Gated single-photon emission computed tomography myocardial perfusion imaging (GSPECT MPI). Among 98 patients with standard resting GSPECT MPI included in this study, 29 received CRT therapy and 69 did not (also had CRT inclusion criteria but did not receive treatment yet at the time of data collection, or refused treatment). A total of 69 non-CRT patients were employed for training, and the 29 were employed for testing. The models were built utilizing features from three distinct feature sets (ConQuaFea, radiomics, and ConQuaFea + radiomics (combined)), which were chosen using Recursive feature elimination (RFE) feature selection (FS), and then trained using seven different machine learning (ML) classifiers. In addition, CRT outcome prediction was assessed by different treatment inclusion criteria as the study’s final phase. The MLP classifier had the highest performance among ConQuaFea models (AUC, SEN, SPE = 0.80, 0.85, 0.76). RF achieved the best performance in terms of AUC, SEN, and SPE with values of 0.65, 0.62, and 0.68, respectively, among radiomic models. GB and RF approaches achieved the best AUC, SEN, and SPE values of 0.78, 0.92, and 0.63 and 0.74, 0.93, and 0.56, respectively, among the combined models. A promising outcome was obtained when using radiomic and ConQuaFea from GSPECT MPI to detect left ventricular contractile patterns by machine learning.

**Supplementary Information:**

The online version contains supplementary material available at 10.1007/s10278-022-00705-9.

## Introduction

Heart failure (HF) is a relatively common cardiovascular disorder with prominent morbidity and mortality [1]. HF is closely related to left ventricular (LV) cardiac mechanical dyssynchrony, which reflects timing differences across various left ventricle regions [2]. Nonhomogeneous contraction patterns can be caused by the uncoordinated distribution of electrical activation in the heart pathways, known as cardiac dyssynchrony [3–5]. Thus, therapeutic measures are developed to resynchronize the left ventricular contraction in HF patients.

Cardiac resynchronization therapy (CRT) demonstrated significant success in treating patients with fatal HF [6, 7]. Patients undergoing CRT have substantiated the claims of most previous studies regarding the enhancements in several parameters, such as 6-min walking distance, New York Heart Association (NYHA) functional class, quality of life score, and peak O_2_ [6]. Nonetheless, estimations demonstrated that approximately one-third of chosen cases do not respond to this costly and invasive therapy [8–12] in spite of meeting current inclusion criteria for CRT therapy by the guidelines, which includes NYHA III or IV, left ventricular ejection fraction (LVEF) < 35%, and QRS duration ≥ 130 ms [13]. Consequently, the search for more specific criteria is still under investigation.

In line with finding new and more appropriate indicators for CRT patient selection, several studies have been conducted. In a study by Bax et al. [14], LV dyssynchrony was introduced as a desirable indicator. Furthermore, in another study conducted by Chen et al. [15], histogram bandwidth and phase standard deviation parameters extracted from phase analysis were suggested as important indicators. In addition, in examining the locations of CRT leads by Adelstein et al. [9], it was found that the best locations are far away from the scar tissue [9]. New LV mechanical dyssynchrony parameters were extracted from GSPECT MPI phase analysis with deep learning to aid CRT patient selection by He et al. [16].

Two types of left ventricular contraction patterns including U-shaped and non-U-shaped patterns have been recently proposed by different imaging modalities [17]. A U-shaped pattern is formed by a left ventricle–directed linear blockage which impedes contraction propagation [18, 19]. Conversely, a non-U-shaped pattern consists of two types, namely, “homogenous contraction” with apparent delay on all walls and “heterogeneous contraction” with multiple contraction delays in different sites [19]. An improved CRT response is discerned to be in association with a U-shaped contraction pattern [17–22].

Myocardial perfusion imaging (MPI) with gated single-photon emission computed tomography (GSPECT) is a practical technique to assess perfusion and function of left ventricle (LV). Evaluation of LV dyssynchrony and LV contraction patterns can also be ascertained by applying phase analysis to GSPECT MPI. The advantage of simultaneous assessment of perfusion abnormalities, such as the extent and severity of ischemia and scar, and functional parameters including LV dyssynchrony, LVEF, and LV volumes make this modality an eligible method for evaluation and diagnosis of patients with HF and LV dysfunction. Besides, the automated nature of phase analysis results in an acceptable repeatability and reproducibility of this technique. As a result, MPI GSPECT is a relevant modality for examining the left ventricular contractile pattern [19, 23].

Recent studies concerning quantitative radiomics analysis, through acting as biomarkers, have provided new insights into better handling of diseases, such as cancer [24] and coronary artery disease (CAD) to predict survival [25, 26], prognosis [27, 28], and therapeutic response [29, 30], different pathology classification [28, 31–33], and accumulating data for personalized medicine [34]. In fact, radiomics is an almost new science that has attracted many researchers’ attention and is therefore growing rapidly. However, it is not yet fully prepared to enter the clinical phase. In fact, radiomics analysis has been performed on magnetic resonance imaging (MRI), computed tomography (CT), and positron emission tomography (PET) images, but less on SPECT owing to its low spatial resolution and low sensitivity. It should be noted that in recent studies, radiomics has performed well in brain SPECT images as well as heart SPECT images [35–37]. Radiomic features are used to feed machine learning algorithms. Machine learning techniques, through the usage of computer algorithms and advanced statistical techniques, facilitate automatic extraction of prognostic knowledge or discriminatory patterns from data, often with the aim of making prediction on new data [38–40].

In this work, the purpose of automatic detection of left ventricular contractile patterns is fulfilled by radiomic and conventional quantitative features (ConQuaFea) using machine learning algorithms and MPI GSPECT images. This study aims to help clinicians to more confidently select patients who are eligible for CRT treatment and will also save them time and energy. In the last step, a comparison was made with the evaluation of response to CRT treatment using the left ventricular contraction pattern diagnosed by two experienced nuclear medicine physicians and the models presented in this study and standard criteria for prescribing CRT.

## Materials and Methods

**Fig. 1:**
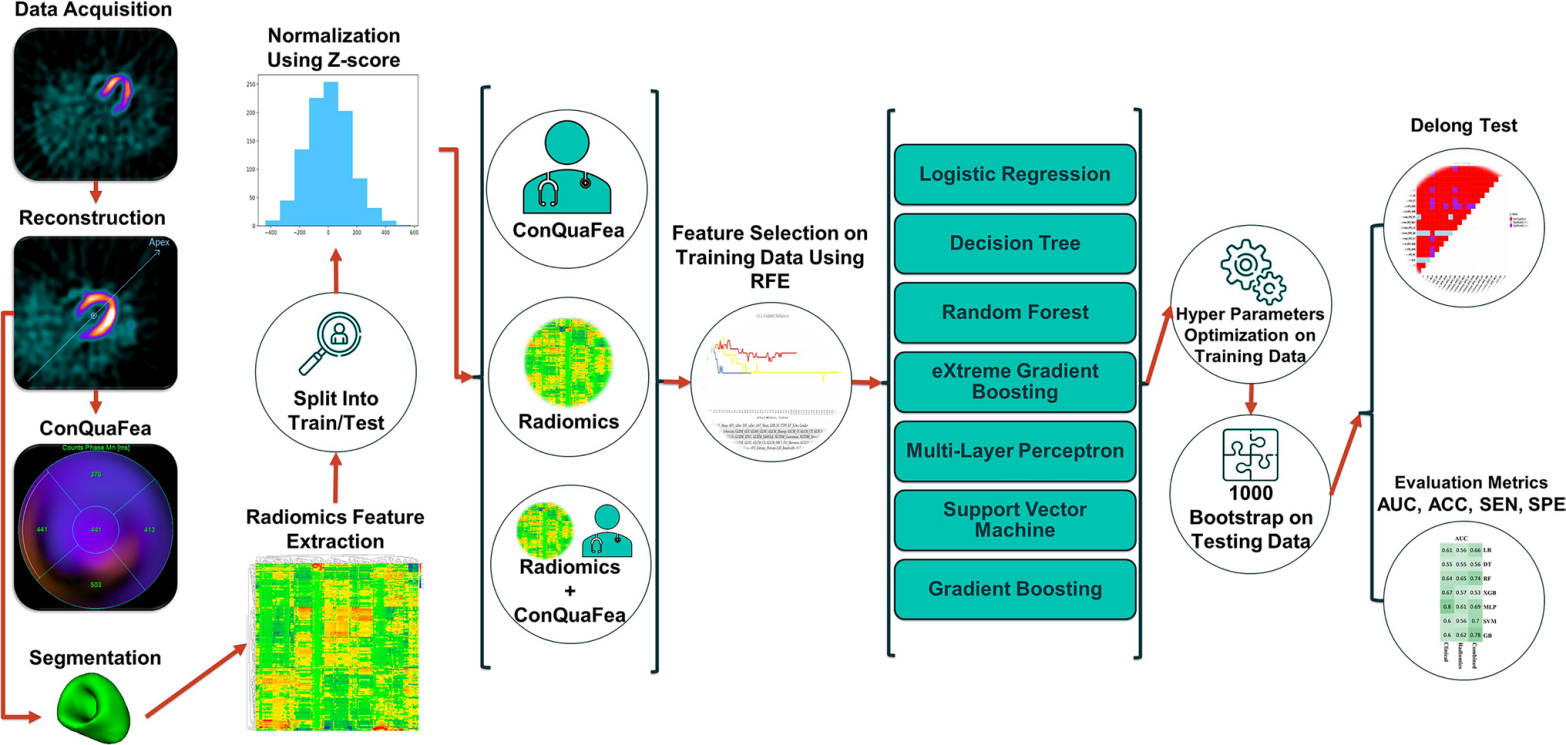
Flowchart describing the main steps involved in the presented study

Figure 1 represents the graphical pipeline of the study. The whole workflow of the study, from data acquisition to evaluation of the proposed models, is elaborated in the following sub-sections as shown in Fig. 1.

### Dataset and Image Acquisition

This is a retrospective study including 98 patients encompassing 29 patients who underwent CRT treatment and 69 patients who did not (not being treated yet at the time of data collection or refused treatment) but had the same inclusion criteria: (a) EF ≤ 35% based on echocardiography and (b) QRS duration ≥ 130 ms [13]. Eighty-three men and 15 women (mean age = 59.37) were selected as participants. Out of 98 patients, 48 had U-shaped and 50 had non-U-shaped contractile patterns (visually assessed by two experienced nuclear medicine physicians from the polar maps as described in detail in the following section). Overall, among the patients who underwent CRT, 13 had U-shaped and 16 had non-U-shaped patterns, and among those patients who did not undergo CRT, 35 had U-shaped and 34 had non-U-shaped patterns. All patients underwent MPI GSPECT with the same vendor and same acquisition parameters. Each patient registered, underwent conventional resting MPI GSPECT. At rest condition, 555–740 MBq of Tc-99 m sestamibi was intravenously administered, and the GSPECT scan started 45–90 min post-injection. The images were acquired on a dual-headed gamma camera (Symbia™ T2, Siemens Healthcare) with body auto-contour form 135° (RAO) to − 45° (LAO) in 180° orbit, 32 thirty-second steps and 16-bin gating, using a matrix size of 64 × 64 with an isotropic voxel size (6.591 × 6.591 × 6.591 mm^3^) in the reconstructed images. The photopeak was adjusted to 140 keV with 20% energy window. Image reconstruction was accomplished using filtered backprojection technique with a post-reconstruction Butterworth filter (order = 5, cut-off frequency of 0.45 cycles/mm).

### Conventional Quantitative Features (ConQuaFea)

Phase analysis was also performed using quantitative gated SPECT (QGS) software (Table 1) to extract several quantitative image-based features. These are summarized in Table 1, “Features extracted from Quantitative Gated SPECT (QGS)” section. Furthermore, other categorical image-based features, such as perfusion, phase, and wall motion, were also collected from polar maps (Table 2). In addition, explanations related to the phase analysis and QGS features are provided in Supplementary Table A.1. The results report the mean and interquartile range (IQR). The *p* values were calculated for continuous features using *t*-test and for categorical features using chi-square. Moreover, the way of scoring different regions of the heart is available in Table 3 according to [41]. Quantitative image-based and phase analysis data were combined and are reported as conventional quantitative features (ConQuaFea) set in the rest of the manuscript.

**Table 1:** First part of ConQuaFea including phase analysis and QGS features, separately for cohorts of patients with U-shaped and non-U-shaped contractile patterns. *IQR* interquartile range

		**ConQuaFea first part**	**Non-u-shaped (*n* = 50)** **Mean and IQR**	**U-shaped (*n* = 48)** **Mean and IQR**	***P* value**
		**Age (years)**	59.52 and 13.00	59.23 and 13.25	0.070
		**Ejection fraction (Echo) (%)**	29.16 and 10.00	30.10 and 10.25	0.269
Phase analysis indices	Apex	Bandwidth (ms)	125.20 and 114.00	128.63 and 114.00	0.754
		Mean	356.32 and 88.00	340.50 and 86.25	0.478
		Standard deviation	35.70 and 35.00	37.96 and 35.00	0.947
		Entropy (%)	39.11 and 33.90	39.51 and 34.03	0.535
	Lateral	Bandwidth (ms)	123.70 and 104.00	164.73 and 112.50	**0.018**
		Mean	340.46 and 78.00	368.58 and 78.50	0.755
		Standard deviation	35.30 and 27.00	46.96 and 30.25	**0.032**
		Entropy (%)	45.87 and 18.80	48.06 and 19.43	0.134
	Inferior	Bandwidth (ms)	121.88 and 93.00	143.92 and 92.25	0.523
		Mean	341.10 and 59.00	363.38 and 56.75	**0.047**
		Standard deviation	34.24 and 27.00	41.46 and 27.50	0.395
		Entropy (%)	46.97 and 17.40	49.02 and 17.15	0.723
	Septal	Bandwidth (ms)	134.96 and 118.00	147.71 and 116.00	0.476
		Mean	344.86 and 78.00	350.96 and 78.75	0.138
		Standard deviation	38.84 and 35.00	40.33 and 34.25	0.981
		Entropy (%)	47.40 and 19.60	45.60 and 19.70	0.941
	Anterior	Bandwidth (ms)	131.54 and 116.00	159.21 and 114.75	0.301
		Mean	333.36 and 57.00	350.90 and 57.00	0.288
		Standard deviation	36.40 and 35.00	43.60 and 34.50	0.312
		Entropy (%)	46.59 and 20.80	50.07 and 20.35	0.864
Features extracted from quantitative gated SPECT (QGS)	Lung heart ratio		0.36 and 0.11	0.40 and 0.11	0.155
	Summed motion score		27.00 and 20.00	25.90 and 19.25	**0.000**
	Summed thickening score		18.26 and 16.00	18.10 and 14.50	**0.004**
	Summed motion (%)		31.74 and 23.00	30.46 and 22.25	**0.000**
	Summed thickening (%)		35.72 and 31.00	35.46 and 29.50	**0.004**
	End-diastolic volume (QGS) (ml)		180.20 and 80.00	153.27 and 80.75	0.076
	End systolic volume (QGS) (ml)		123.68 and 68.00	98.94 and 67.50	**0.032**
	Systolic volume (ml)		56.80 and 26.00	54.29 and 26.00	0.732
	Ejection fraction (%)		37.02 and 17.00	37.81 and 17.00	**0.030**
	Peak emptying rate (EDV/s)		− 2.07 and 1.04	− 1.99 and 0.99	0.181
	Peak filling rate (EDV/s)		1.44 and 0.85	1.72 and 0.85	0.594
	Peak filling rate2 (EDV/s)		1.39 and 0.97	1.00 and 0.98	0.243
	Mean filling rate/3 (EDV/s)		0.74 and 0.60	0.84 and 0.55	0.866
	Time to peak filling from ES (ms)		138.25 and 80.00	159.56 and 79.50	0.914
	Beats per minute (beats/minute)		773.20 and 224.00	856.54 and 235.50	0.788

**Table 2:** Second part of ConQuaFea including wall motion, phase, and perfusion features, separately for cohorts of patients with U-shaped and non-U-shaped contractile patterns

ConQuaFea second part			Non-u-shaped	U-shaped	*P* value
**Wall motion**	Septal	# in classes (0, 1, 2, 3, 4, 5)	2, 3, 8, 7, 10, 20	7, 4, 9, 5, 14, 9	0.150
	Anterior		16, 7, 11, 5, 10, 1	17, 6, 11, 10, 3, 1	0.357
	Lateral		19, 8, 9, 10, 3, 1	22, 7, 10, 6, 3, 0	0.806
	Inferior		10, 8, 8, 9, 13, 2	14, 4, 9, 7, 13, 1	0.761
	Apex		11, 6, 6, 7, 11, 9	10, 3, 7, 2, 18, 8	0.346
**Phase**	Septal	# in classes (1, 2, 3, 4, 5)	9, 11, 8, 9, 13	10, 7, 11, 8, 12	0.831
	Anterior		12, 13, 10, 12, 3	11, 14, 11, 9, 3	0.972
	Lateral		6, 12, 12, 8, 12	11, 10, 5, 9, 13	0.331
	Inferior		9, 4, 14, 16, 7	5, 9, 8, 16, 10	0.268
	Apex		17, 9, 6, 4, 14	10, 11, 14, 4, 9	0.180
**Perfusion**	Septal	# in classes (0, 1, 2, 3, 4)	28, 10, 4, 6, 2	31, 6, 4, 4, 3	0.788
	Anterior		33, 3, 9, 3, 2	33, 6, 3, 3, 3	0.385
	Lateral		38, 6, 2, 3, 1	38, 5, 4, 1, 0	0.606
	Inferior		26, 8, 8, 6, 2	29, 11, 5, 3, 0	0.368
	Apex		20, 7, 5, 8, 10	26, 4, 3, 5, 10	0.600

**Table 3:** Scoring heart segments in terms of wall motion, perfusion, and phase

Categorical features	Scores
Wall motion	0 = normal 1 = mild hypokinesia 2 = moderate hypokinesia 3 = severe hypokinesia 4 = akinesia 5 = dyskinesia
Perfusion	0 = normal 1 = mild hypoperfusion 2 = moderate hypoperfusion 3 = severe hypoperfusion 4 = absent perfusion
Phase	1 = fastest contraction 5 = slowest contraction

### Investigation of Myocardial Contractile Patterns from Polar Maps

The myocardial uptake is divided into a limited number of raw perfusion samples after the left ventricle has been segmented, each represented by the mean or maximal wall photon counts at a specific place on the myocardial surface [42]. The objective is to parametrically represent myocardial perfusion to enable standard inter-subject comparability. A visual representation known as a “polar map” or “bull’s eye map” was created to make it easier to analyze the data obtained by polar sampling [43]. In clinical practice, polar maps are often employed because they provide a fast visual overview of myocardial perfusion data for the whole LV. Additionally, polar maps enable the myocardium to be divided into sections defined by vascular regions, LV wall, normalized 21-segment, 17-segment, or 5-segment status, which are useful for localizing the abnormalities [44, 45].

**Fig. 2:**
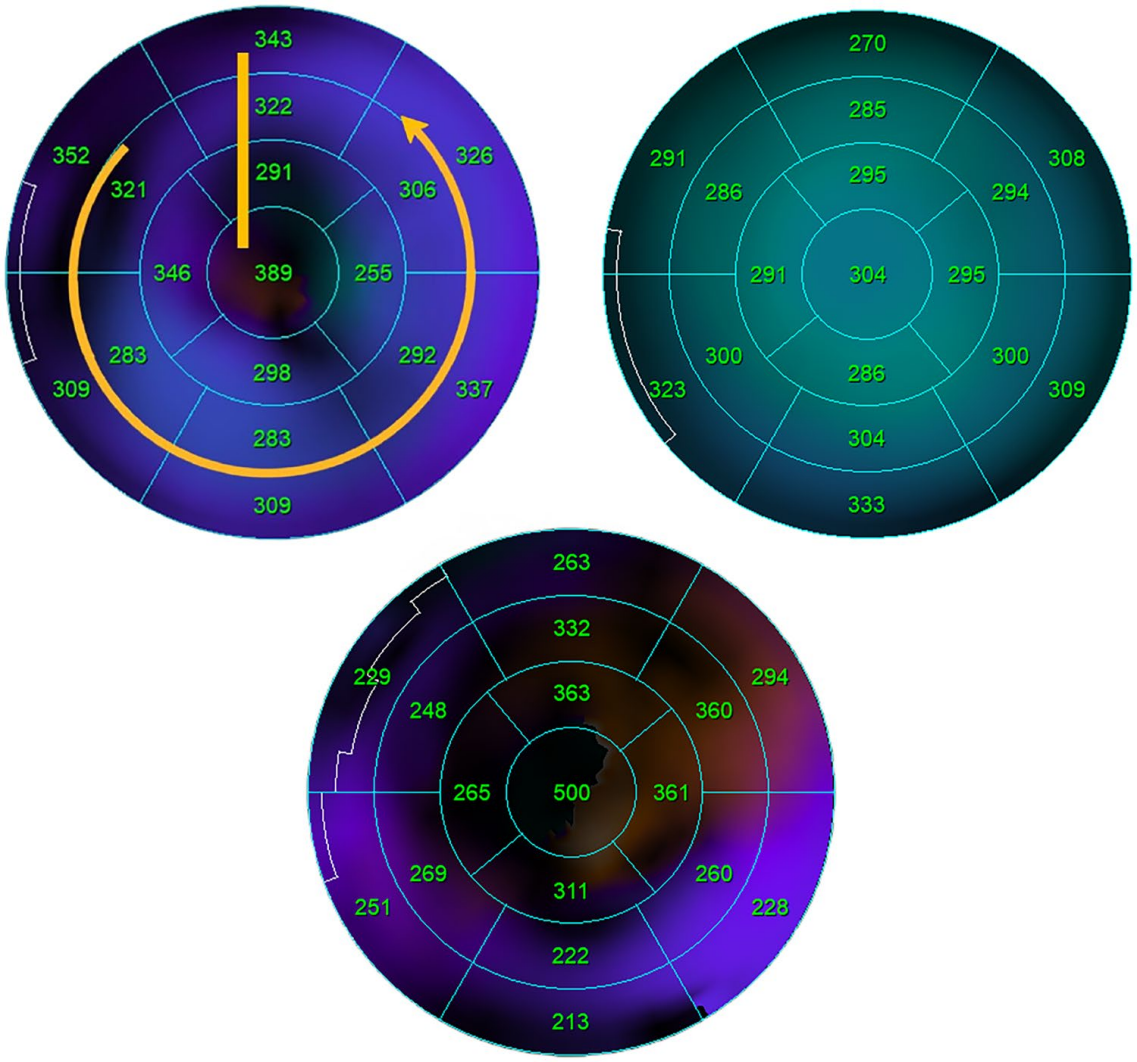
Illustration of different contraction patterns of the left ventricle showing U-shaped (upper right), non-U-shaped (upper left) (homogeneous), and non-U-shaped (heterogeneous) (bottom)

Mechanical left ventricular dyssynchrony can produce different patterns in polar map images as a result of two types of electrical activity propagation in the left ventricular myocardium, which include a U-shaped pattern and a non-U-shaped pattern. U-shaped pattern means that electrical activity propagation is blocked in a line while the other walls are almost contracted. For example, a contraction of an area starts from the anteroseptal wall and then goes to the apex, the lateral wall, and finally to the anterior wall. However, there are two types of non-U-shaped pattern. The first type is a homogeneous pattern; for example, contraction goes from the septal wall to the lateral wall. The second type is the heterogeneous pattern, in which there are multiple areas for myocardial contraction [19]. Different contraction patterns of the left ventricle can be seen in Fig. 2. In this study, the contraction pattern was assessed visually by two nuclear medicine physicians from the 17-segment mode polar maps polar maps.

### Radiomics Feature Extraction

To extract MPI SPECT radiomic features, the left ventricle was segmented by an experienced nuclear medicine technologist and edited/verified by a nuclear medicine physician. Then, feature extraction was conducted utilizing pyradiomics, a library in python compliant with image biomarker standardization initiative (IBSI) [40, 46]. Re-sampling was performed for all images with order 3 interpolation using sitkBSpline to 6.591 × 6.591 × 6.591 mm^3^ voxels. Intensities within the volume of interest (VOI) were separated to 32 discrete gray levels with fixed bin number technique. A total of 107 radiomic features including shape, intensity, and second-/high-order texture features from GLDM, GLCM, GLRLM, GLSZM, and NGTDM families were extracted. The name and short description of all radiomic features are presented in Supplementary Table A.2.

### Machine Learning Workflow

As can be seen in Fig. 1, after the feature extraction process, the data was split into train/test partitions in a way that 69 non-CRT patients were used for training and 29 CRT patients were used for testing. In addition, three feature sets were pursued for modeling. In the first case, only ConQuaFea was used for modeling. In the second case, only radiomic features and finally, in the third case, a combination of these two feature sets (combined) were used for modeling. In all models, the features extracted from the training dataset were normalized using Z-score method, and the calculated mean and standard deviation (SD) were applied on corresponding features extracted from the test dataset.

The feature selection method used in this study was recursive feature elimination (RFE). Modeling was performed using logistic regression (LR), decision tree (DT), random forest (RF), extreme gradient boosting (XGB), multi-layer perceptron (MLP), support vector machine (SVM), and gradient boosting (GB) algorithms. As a result, a total of 21 different models (3 kinds of feature-sets including ConQuaFea, radiomics, and combined × 1 feature selection method × 7 machine learning methods) were implemented. Hyper-parameters were optimized using GridSearch with 5-fold cross-validation in training data, and best values were selected to train model followed by applying the trained model on test data by 1000 bootstrap. The best hyper-parameters for each classifier are presented in Table A.3. Area under the ROC curve (AUC), accuracy (ACC), sensitivity (SEN), and specificity (SPE) metrics were used to evaluate the models. All analysis was performed on Python 3.8.8.

### Delong Test

Using Delong test, comparisons between AUC of all models were performed, which was in turn followed by false discoveries rate (FDR) correction with Benjamini–Hochberg method applied on *p* values. Consequently, adjusted *p* values, also known as *q* values, were assessed. *P* values less than 0.05 were considered statistically meaningful.

### Evaluation of CRT Response

In the final step of the study, the task of differentiating between patients responding to CRT or not was assessed by different inclusion criteria for the treatment, namely, (1) conventional criteria and (2) left ventricle contractile pattern status assessed visually by two nuclear medicine physicians or alternatively by machine learning and ConQuaFea/radiomic features. These patients considered as responders to CRT, experienced an improvement of at least 5% in LVEF during six months of follow-up [13].

## Results

### Selected Features

**Fig. 3:**
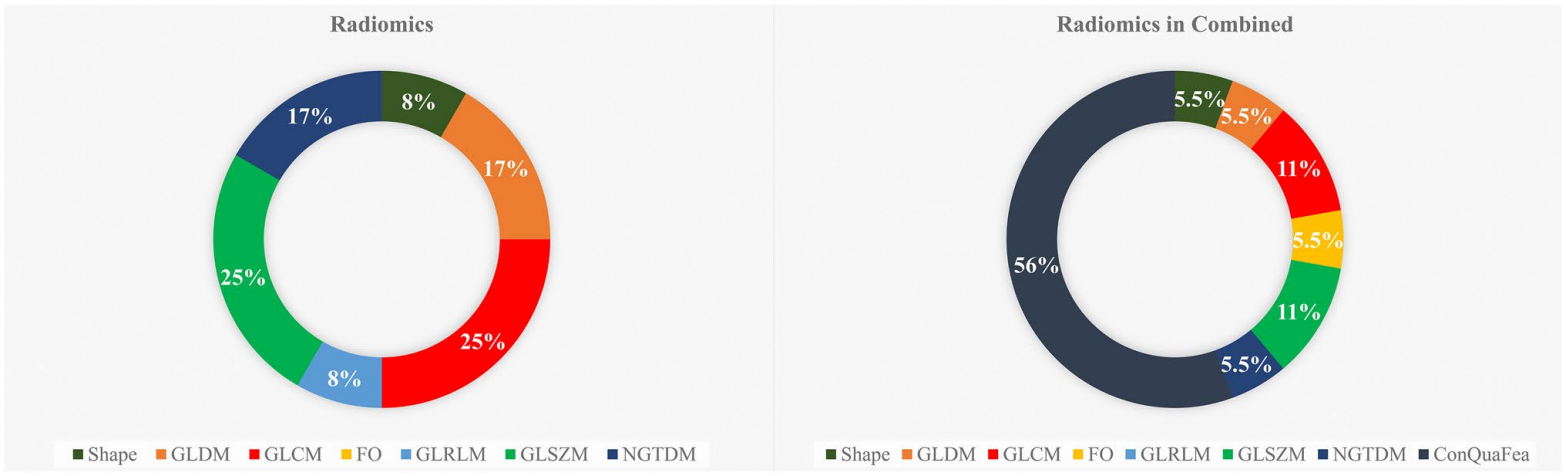
Distribution of selected radiomic features by RFE feature selection method across different feature families. The left chart is related to the time when the features were selected from the radiomic data set, whereas the right chart is related to the time when the radiomic features were selected from combined features

Based on RFE feature selection, 9 ConQuaFea, 18 combined, and 12 radiomic features were selected. Figure 3 illustrates the distribution of selected radiomic features over different feature families in the form of a pie chart. As it is shown in Fig. 3, GLCM and GLSZM features were selected the most, following them were GLDM and NGTDM which had the highest selection. This is while, first order in radiomic features and GLRLM in combined features were not selected by the feature selection methods.

**Fig. 4:**
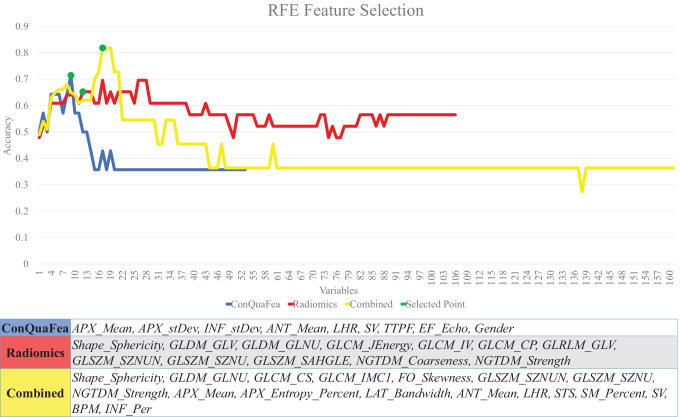
The process of RFE feature selection and the resultant features. The green circles show the selected points (APX_Mean, apex mean; APX_stDev, apex standard deviation; INF_stDev, inferior standard deviation; ANT_Mean, anterior mean; LHR, lung heart ratio; SV, systolic volume; TTPF, time to peak filling from ES; EF_Echo, ejection fraction (Echo); APX_Entropy_Percent, apex entropy (%); LAT_Bandwidth, lateral bandwidth; STS, summed thickening score; SM_Percent, summed motion (%); BPM, beats per minute; INF_Per, inferior perfusion)

Figure 4 illustrates the selection process and lists the selected features by RFE feature selection for radiomics, ConQuaFea, and combined models.

### Models’ Performance

**Fig. 5:**
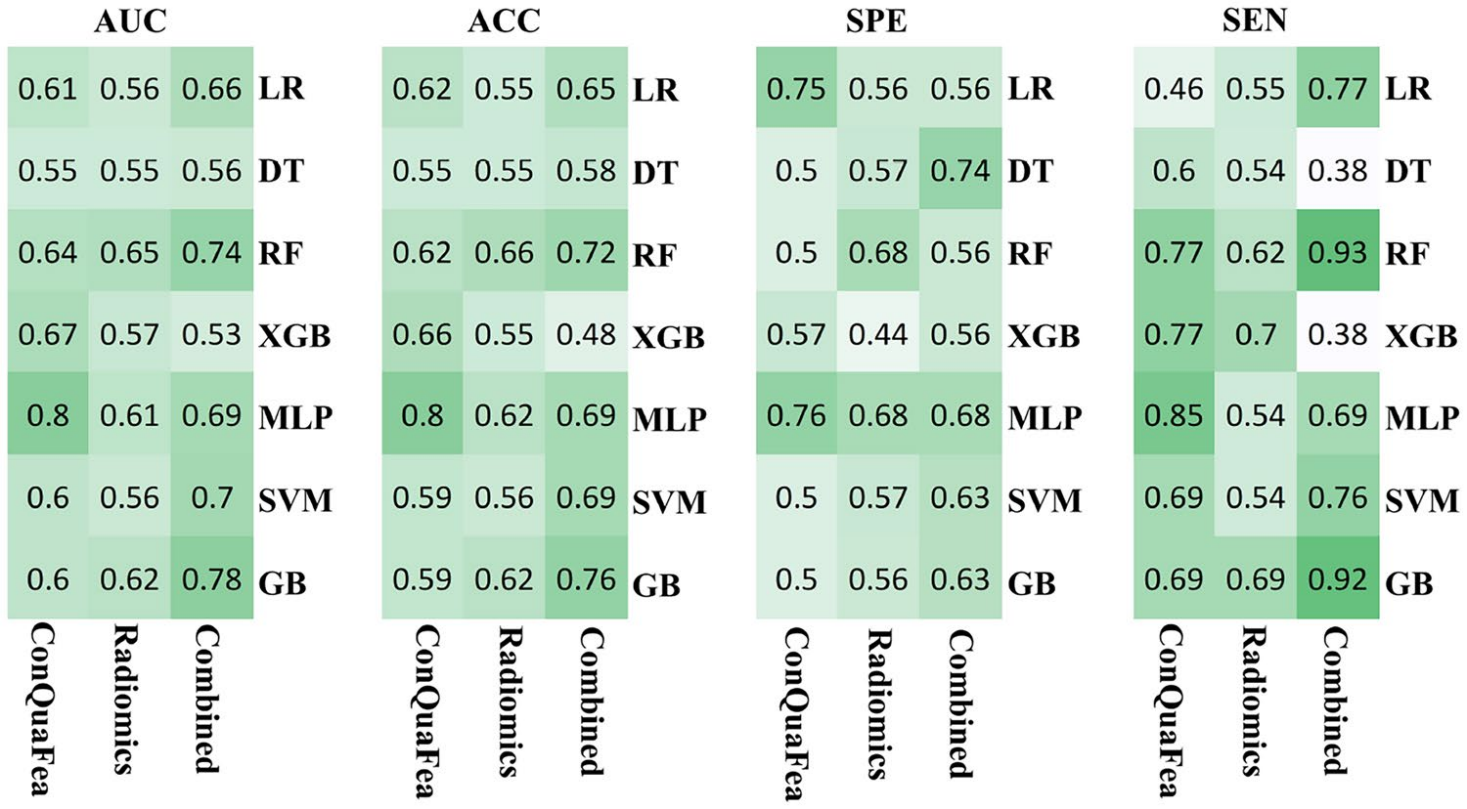
Performance metrics of ConQuaFea, radiomic, and combined features. The metrics include the area under the curve (AUC), accuracy (ACC), sensitivity (SEN), and specificity (SPE). Seven different classifiers with a feature selection method and 3 different feature sets are considered

The models were developed using features from three different feature sets (ConQuaFea, radiomics, and combined), selected by RFE feature selection method, and trained with seven different machine learning methods (LR, DT, RF, XGB, MLP, SVM, and GB). Figure 5 illustrates performance metrics of all models. The performance metrics reported in Fig. 5 include AUC, accuracy, sensitivity, and specificity.

The best performance among ConQuaFea models was achieved by MLP classifier (ACC, AUC, SEN, SPE = 0.80, 0.80, 0.85, 0.76, respectively). Among radiomics models the best models was RF model (ACC, AUC, SEN, SPE = 0.66, 0.65, 0.62, 0.68, respectively). Among the combined models, the best ones were GB and RF machines (ACC, AUC, SEN, SPE were 0.76, 0.78, 0.92, 0.63, and 0.72, 0.74, 0.93, 0.56, respectively).

**Fig. 6:**
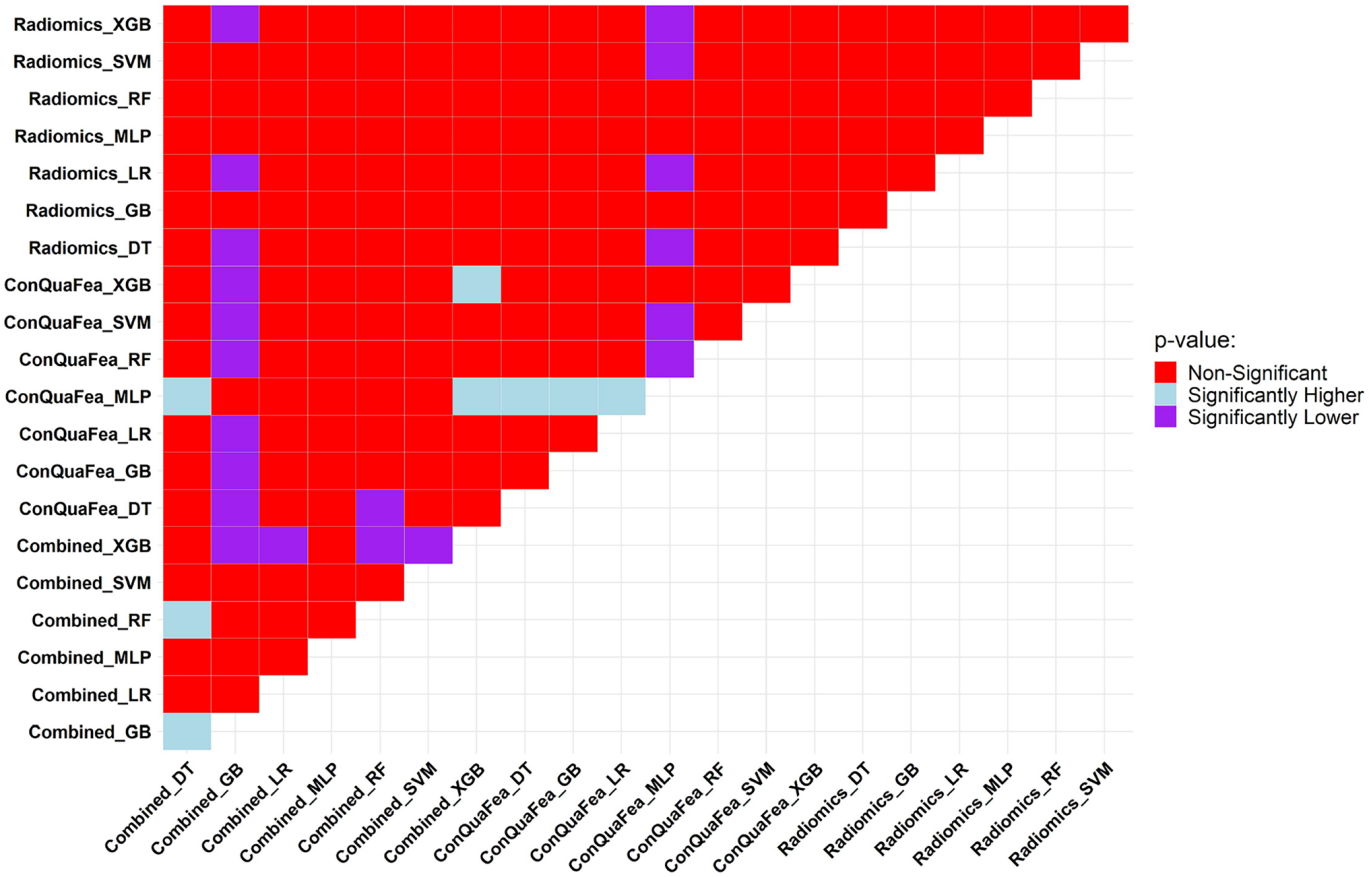
Comparison of model’s performance through the Delong test being applied on the AUCs of models. We compare the pairwise model in this figure where the row models were tested against column models. Light blue, if the row model had significantly higher *p* value than the column model; purple, if the row model had significantly lower *p* value compared to the column model; red, if the comparison between the row model and column model had non-significant *p* value

The results of the Delong test are illustrated in Fig. 6. The AUC of each model was compared with 20 other models. The results were classified as statistically significant (significantly lower or significantly higher) and non-significant. In Fig. 6, the MLP classifier on ConQuaFea had the best results with 11 significantly higher *q* values. Regarding radiomic features, the RF classifier did not have any significant *q* values. In terms of models with combined feature sets, GB and RF classifiers had 11 and 3 significantly higher *q* values, respectively.

### CRT Response Prediction

The primary aim of this study was to predict contractile patterns using GSPECT MPI, but the performance of the different models regarding the prediction of CRT response for 29 patients undergoing the treatment were also evaluated. Table 4 illustrates outcomes of 29 patients who underwent CRT with the confusion matrix regarding the prediction of models based on conventional criteria and myocardium contractile pattern (identified by two nuclear medicine physicians and different proposed machines) for prescription of the treatment. Table 4 is color coded as blue for correct decisions (true positive and negative) and red for wrong decisions (false positive and negative). The first row of Table 4 shows that based on the conventional criteria, from 29 patients prescribed with CRT, only 16 patients (55%) responded positively after the treatment whereas 13 other patients (45%) failed in treatment. The second row shows the scenario in which the treatment criteria was set as the contractile pattern statue (identified by humans). In this case, 13 patients were identified with U-shaped pattern, supposed to be sent for treatment, and if so, only 2 of them would have failed. In addition, from the 16 patients which were identified as non-U-shaped, they were not supposed to be sent for treatment, 5 of them would have responded positively to the treatment. The following rows (identified by machine) can be seen in Table 4.

**Table 4:**
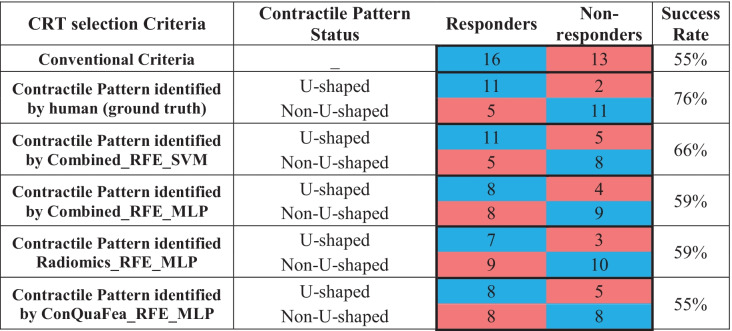
Prediction of models based on conventional criteria and myocardium contractile pattern (identified by two nuclear medicine physicians and different proposed machines) for the prescription of treatment

## Discussion

Echocardiography, non-contact mapping (NCM), cardiac magnetic resonance (CMR), and myocardial perfusion imaging (MPI) are discovering two types of contraction patterns in the left ventricle. NCM is considered the gold standard of assessing the patterns of LV electrical activation. Yet the hazardous and invasive nature of the procedure restricts its wide clinical application [18]. Accessibility, noninvasiveness and low cost are the advantages leading to wide usage of echocardiography. However, low repeatability of echo-based parameters, the state of being operator-dependent and suboptimal acoustic window for 20% of the patients have led to the approach being unpromising in selecting patients for CRT [52, 53].  CMR has emerged as a valuable tool to assess LV contraction patterns due to its high resolution and excellent tissue characterization which make it a promising approach for selecting responsive patients to CRT. Nevertheless, a high percentage of patients are disqualified on account of having pacemakers and implantable cardioverter defibrillators (ICDs) or as a result of being claustrophobic. In addition, the procedure is expensive, difficult to access, and extensively time-consuming [52–54]. GSPECT MPI transpires to be a practical technique to ascertain LV contraction patterns. Furthermore, GSPECT MPI is considerably being used for HF patients to identify LV dyssynchrony, LVEF, LV volumes, ischemia, viability, and scar tissue. Moreover, the advantages include the state of being ubiquitous and automated [19, 23, 49].

To the best of our knowledge, our study represents the first attempt to label left ventricular contractile patterns by ConQuaFea and radiomic features using machine learning and GSPECT MPI images. While this task has been previously studied in GSPECT MPI [19], it has not been investigated by machine learning approaches as well as radiomic features. In this study, we applied multiple machine learning and a feature selection method on feature sets, including radiomics, ConQuaFea/phase-analysis, and combination of both features to develop machines for the identification of LV contractile pattern from the MPI GSPECT images.

Toward the identification of most relative radiomic features, Fig. 3 illustrates the distribution of radiomic features selected by RFE feature selection algorithm over radiomic feature families. As it can be seen, GLCM and GLSZM features followed by GLDM and NGTDM were the most selected features, respectively, showing the highest correlation of the texture, hence the heterogeneity of the underlying biology of the myocardial tissue with the outcome of interest. First-order radiomic features and GLRLM in combined features were not selected by RFE. In addition, in both cases, only one feature in shape family was selected. Since the region of interest was set as the whole left ventricle, it was expected that morphological features do not show much correlation with the desired outcome.

Toward the identification of the optimum automated model, different combinations of RFE feature selection and machine learning algorithms were applied on the included feature sets (ConQuaFea, radiomics, and combined). ConQuaFea feature sets showed superiority over radiomic features. Nevertheless, radiomic features also showed an adequate performance. However, it was suggested that further studies should investigate the correlations between different feature sets in an independent analysis.

In summary, our study highlighted the potential of MPI SPECT ConQuaFea. Radiomic features for the identification of left ventricular contractile patterns also showed an acceptable performance. Physicians may benefit from evaluating each patient’s specific condition and determine if CRT is needed. Moreover, it can reduce the workload and time spent by physicians, since the detection of U-shaped contractile patterns takes an average of 5 min per patient. In addition, excessive workloads, long working hours, sleep deprivation, expertise of the physician, and other factors can lead to misdiagnosis. Hence, this study attempts to help physicians making better and more precise decisions in short time.

As mentioned earlier, despite the effectiveness and importance of CRT [6, 7], at least 30% of patients selected for this treatment do not respond well to this costly and invasive treatment [8–12], showing insufficiency of current inclusion criteria for this treatment. Moreover, from the 29 patients treated with CRT, 13 patients (45%) did not respond to the treatment (Table 4). A number of studies attempted to introduce new criteria to improve the response rate to treatment. In Bax et al*.* [14, 47], the presence of left ventricular mechanical dyssynchrony was more common in patients responding to CRT treatment, and therefore, it was suggested as a criterion to improve response to CRT treatment. A study by Adelstein et al*.* [9] also suggested that CRT leads should not be placed in areas of scar tissue diagnosed by MPI prior to implantation, as otherwise the response to treatment will be reduced. In the study of Chen et al. [15], the authors realized that the only characteristics which were different between the CRT patients who were responders and those who were not, were the histogram bandwidth and phase standard deviation (extracted from phase analysis using Emory Cardiac Toolbox at the baseline (before CRT)), which is related to left ventricular mechanical dyssynchrony. He et al. [16] conducted research with the goal of using deep learning to extract new LVMD features using GSPECT MPI phase analysis to help selecting CRT patients. New LVMD parameters retrieved by automated Autoencoder (AE) from GSPECT MPI have the potential to enhance response prediction prior to CRT. The study by Rastgou et al. [48] stated that the phase analysis parameters of Emory Cardiac Toolbox and QGS program are well correlated, but these parameters should not be used interchangeably. Furthermore, in relation to entropy (a parameter in phase analysis), the lower value of this parameter indicates synchronized heart contraction whereas the higher value indicates desynchronize heart contraction [49].

In our study, the QGS program was used to extract phase analysis indices, and when the features were selected by RFE, the apex standard deviation and inferior standard deviation from ConQuaFea data set and apex entropy and lateral bandwidth from combined features were selected. Since these features are related to the left ventricular contractile pattern and the left ventricular contractile pattern to the type of CRT response, the phase analysis parameters, as mentioned in the above studies, are related to the type of CRT response.

In a study by Feeny et al*.* [50], a machine learning model was developed for CRT outcome prediction by enrolling 925 patients, with 9 clinical features (QRS morphology, QRS duration, New York Heart Association classification, left ventricular ejection fraction and end-diastolic diameter, sex, ischemic cardiomyopathy, atrial fibrillation, and epicardial left ventricular lead) used as input for the Naïve Bayes classifier. Their machine learning model outperformed the conventional guideline with an increased AUC, 0.70 vs. 0.65 (*p* value < 0.02), and an increased event-free survival with concordance index = 0.61 vs. 0.56 (*p* value < 0.001).

In a study by Tao et al*.* [19], contractile patterns were used to analyze CRT responses. Their results showed that 89% of U-shaped group were responders to CRT and 11% were non-responders. In our study, of the 29 patients who performed CRT, 16 patients had U-shaped contractile patterns, from which 11 responded positively, and 13 had non-U-shaped contractile patterns, among which 11 did not respond to the treatment (Table 4). It led to a success rate of 76% compared to traditional patient selection (16 responders out of 29 (55% responders)) in predicting treatment outcome based on contractile patterns. We also analyzed the treatment response, in case CRT was prescribed based on the left ventricle contractile pattern detected by our proposed models (Table 4). The results were slightly lower than the model based on the ground truth status of the left ventricle contractile pattern identified by the two nuclear medicine physicians which was expected since the error of pattern identification is also introduced to the final results. Moreover, it should be noted that according to the study conducted by Hartlage et al*.* [17], LV lead concordant to the latest contracting site would be more likely to produce a superior CRT response beside the left ventricular contractile pattern. In fact, in their study, patients with a U-shaped contraction pattern and the LV lead concordant to the last contraction site were 92% respondent, which indicates the importance of examining the pattern as well as the correct location of the lead in CRT patients. In our study, the main purpose was to detect the left ventricular contractile pattern, and therefore, the correct location of the leads was not investigated.

This study inherently bears a few limitations. Regarding the task of CRT outcome prediction, we set the criteria on the basis of the left ventricular contractile pattern and did not consider matters, such as the location of the CRT leads. Future studies might consider both to develop more comprehensive automated models for this important task. In addition, our dataset was obtained from one institute which undermines the robustness of the findings. Further studies might gather larger and diverse dataset obtained from multiple institutes with different image acquisition parameters and patients’ ethnicity to improve the reproducibility of the models [51]. However, for proof-of-concept, this study contained enough patients.

## Conclusion

In this study, machine learning models were developed to predict left ventricular contractile patterns with ConQuaFea and radiomic features from GSPECT MPI using different machine learning approaches with acceptable and promising results. ConQuaFea performed better than radiomic features in recognizing left ventricular contractile pattern. In addition, by diagnosing the patients’ left ventricular contraction pattern, it is possible to improve the patient’s selection for CRT treatment.

## Supplementary Information

Below is the link to the electronic supplementary material.

Supplementary file1 (DOCX 24 KB)

## Data Availability

Not applicable.

## Abbreviations

ConQuaFea: Conventional quantitative features
HF: Heart failure
CRT: Cardiac resynchronization therapy
NYHA: New York Heart Association
LVEF: Left ventricular ejection fraction
LV: Left ventricle
CAD: Coronary artery disease
GSPECT MPI: Gated single-photon emission computed tomography myocardial perfusion imaging
NCM: Non-contact mapping
CMR: Cardiac magnetic resonance
MRI: Magnetic resonance imaging
CT: Computed tomography
PET: Positron emission tomography
QGS: Quantitative gated SPECT
IBSI: Image Biomarker Standardization Initiative
VOI: Volume of interest
SD: Standard deviation
RFE: Recursive feature elimination
LR: Logistic regression
DT: Decision tree
RF: Random forest
XGB: Extreme gradient boosting
MLP: Multi-layer perceptron
SVM: Support vector machine
GB: Gradient boosting
AUC: Area under the ROC curve
ACC: Accuracy
SEN: Sensitivity
SPE: Specificity
FDR: False discoveries rate

## Code Availability

Open-source library including 3D-Slicer and Python library used in this study.
