# Supplementary material for: Myocardial Perfusion SPECT Imaging Radiomic Features and Machine Learning Algorithms for Cardiac Contractile Pattern Recognition
Source: J Digit Imaging. 2022 Nov 14;36(2):497–509. doi: 10.1007/s10278-022-00705-9 (PMC10039187; doi:10.1007/s10278-022-00705-9)
Supplement: Supplementary file 1 — Supplementary file1 (DOCX 24 KB) [file 10278_2022_705_MOESM1_ESM.docx]

**Supplementary Table A.1.** Definition of various features extracted from QGS programs.

|  | Feature | Definition |
| --- | --- | --- |
| Phase analysis indices | **Bandwidth (ms)** | 95% of the elements of the phase distribution are included in the histogram's bandwidth. |
|  | **Mean** | Average of sampling points over the region. |
|  | **Standard deviation** | Standard deviation of the phase distribution. |
|  | **Entropy (%)** | In histograms, a disordered phase distribution results in an increase in entropy. |
| Features extracted from  Quantitative Gated SPECT (QGS) | **Lung Heart Ratio** | The Lung Heart Ratio (LHR) is obtained by dividing the counts in the left upper lung by the counts over the myocardium. |
|  | **Summed Motion Score** | Summation of the motion visual scores for all segments. |
|  | **Summed Thickening Score** | Summation of the thickening visual scores for all segments. |
|  | **Summed Motion (%)** | Summed motion percent. |
|  | **Summed Thickening (%)** | Summed thickening percent. |
|  | **End Diastolic Volume (QGS) (ml)** | LV chamber volume in ml at end-diastole. |
|  | **End Systolic Volume (QGS) (ml)** | LV chamber volume in ml at end-systole. |
|  | **Systolic Volume (ml)** | The difference between the volume inside the ventricle at the end of diastole and the end of systole. |
|  | **Ejection Fraction (%)** | The volumetric fraction of fluid that is ejected from a chamber with each contraction. |
|  | **Peak Emptying Rate (EDV/s)** | The highest negative velocity in the systolic phase of the time–volume curve. |
|  | **Peak Filling Rate (EDV/s)** | The highest positive velocity during early diastole. |
|  | **Peak Filling Rate2 (EDV/s)** | Secondary peak filling rate. |
|  | **Mean Filling Rate/3 (EDV/s)** | Mean filling rate over the first third of the end-systolic to end-diastolic phase. |
|  | **Time to peak filling from ES (ms)** | Time to peak filling from end-systole. |
|  | **Beats Per Minute (beats/minute)** | Heart rate in heart beats per minute. |

**Supplementary Table A.2.** Name and short description of the extracted radiomic features.

| **Shape** | VoxelVolume: VoxelVolume  M3DD: Maximum3DDiameter  MeshVolume: MeshVolume  MajorAxis: MajorAxisLength  Sphericity: Sphericity  LeastAxis: LeastAxisLength  Elongation: Elongation  SVR: SurfaceVolumeRatio  M2DDS: Maximum2DDiameterSlice  Flatness: Flatness  SurfaceArea: SurfaceArea  MinorAxis: MinorAxisLength  M2DDC: Maximum2DDiameterColumn  M2DDR: Maximum2DDiameterRow | **GLDM** | GLV: GrayLevelVariance  HGLE: HighGrayLevelEmphasis  DE: DependenceEntropy  DNU: DependenceNonUniformity  GLNU: GrayLevelNonUniformity  SDE: SmallDependenceEmphasis  LDHGLE: LargeDependenceHighGrayLevelEmphasis  DNUN: DependenceNonUniformityNormalized  LDE: LargeDependenceEmphasis  LDLGLE: LargeDependenceLowGrayLevelEmphasis  DV: DependenceVariance  SDHGLE: SmallDependenceHighGrayLevelEmphasis  SDLGLE: SmallDependenceLowGrayLevelEmphasis  LGLE: LowGrayLevelEmphasis | **GLSZM** | GLV: GrayLevelVariance  ZV: ZoneVariance  GLNUN: GrayLevelNonUniformityNormalized  SZNUN: SizeZoneNonUniformityNormalized  SZNU: SizeZoneNonUniformity  GLNU: GrayLevelNonUniformity  LAE: LargeAreaEmphasis  SAHGLE: SmallAreaHighGrayLevelEmphasis  ZP: ZonePercentage  LALGLE: LargeAreaLowGrayLevelEmphasis  LAHGLE: LargeAreaHighGrayLevelEmphasis  HGLZE: HighGrayLevelZoneEmphasis  SAE: SmallAreaEmphasis  LGLZE: LowGrayLevelZoneEmphasis  ZE: ZoneEntropy  SALGLE: SmallAreaLowGrayLevelEmphasis |
| --- | --- | --- | --- | --- | --- |
| **First Order** | IQR: InterquartileRange  Skewness: Skewness  Uniformity: Uniformity  Median: Median  Energy: Energy  RMAD: RobustMeanAbsoluteDeviation  MAD: MeanAbsoluteDeviation  TE: TotalEnergy  Maximum: Maximum  RMS: RootMeanSquared  90Percentile: 90Percentile  Minimum: Minimum  Entropy: Entropy  Range: Range  Variance: Variance  10Percentile: 10Percentile  Kurtosis: Kurtosis  Mean: Mean | **GLRLM** | SRLGLE: ShortRunLowGrayLevelEmphasis  GLV: GrayLevelVariance  LGLRE: LowGrayLevelRunEmphasis  GLNUN: GrayLevelNonUniformityNormalized  RV: RunVariance  GLNU: GrayLevelNonUniformity  LRE: LongRunEmphasis  SRHGLE: ShortRunHighGrayLevelEmphasis  RLNU: RunLengthNonUniformity  SRE: ShortRunEmphasis  LRHGLE: LongRunHighGrayLevelEmphasis  RP: RunPercentage  LRLGLE: LongRunLowGrayLevelEmphasis  RE: RunEntropy  HGLRE: HighGrayLevelRunEmphasis  RLNUN: RunLengthNonUniformityNormalized | **GLCM** | JA: JointAverage  SA: SumAverage  JEntropy: JointEntropy  CS: ClusterShade  MP: MaximumProbability  IDMN: Idmn  JEnergy: JointEnergy  Contrast: Contrast  DE: DifferenceEntropy  IV: InverseVariance  DV: DifferenceVariance  IDN: Idn  IDM: Idm  Correlation: Correlation  AC: Autocorrelation  SE: SumEntropy  MCC: MCC  SS: SumSquares  CP: ClusterProminence  IMC2: Imc2  IMC1: Imc1  DA: DifferenceAverage  ID: Id  CT: ClusterTendency |
|  |  | **NGTDM** | Coarseness: Coarseness  Complexity: Complexity  Strength: Strength  Contrast: Contrast  Busyness: Busyness |  |  |

**Supplementary Table A.3.** Information regarding hyper-parameter optimization.

| Feature Sets | Models | Best Parameters |
| --- | --- | --- |
| ConQuaFea | Logistic Regression (LR) | {'C': 1, 'penalty': 'l2', 'solver': 'newton-cg'} |
|  | Decision Tree (DT) | {'criterion': 'gini', 'max_depth': 9, 'max_features': None, 'max_leaf_nodes': 70, 'min_samples_leaf': 4, 'min_weight_fraction_leaf': 0.2, 'splitter': 'random'} |
|  | Random Forest (RF) | {'max_depth': 5, 'max_features': 'sqrt', 'n_estimators': 1000} |
|  | eXtreme Gradient Boosting (XGB) | {'learning_rate': 0.1, 'max_depth': 3, 'n_estimators': 10, 'subsamples': 0.5} |
|  | Multi-Layer Perceptron (MLP) | {'activation': 'relu', 'alpha': 0.05, 'hidden_layer_sizes': (10, 20, 30), 'learning_rate': 'adaptive', 'solver': 'adam'} |
|  | Support Vector Machine (SVM) | {'C': 0.4, 'gamma': 0.5, 'kernel': 'sigmoid'} |
|  | Gradient Boosting (GB) | {'learning_rate': 0.1, 'max_depth': 9, 'n_estimators': 1000, 'subsample': 0.5} |
| Radiomcs | Logistic Regression (LR) | {'C': 10, 'penalty': 'l2', 'solver': 'newton-cg'} |
|  | Decision Tree (DT) | {'criterion': 'gini', 'max_depth': 12, 'max_features': 'auto', 'max_leaf_nodes': 40, 'min_samples_leaf': 2, 'min_weight_fraction_leaf': 0.1, 'splitter': 'best'} |
|  | Random Forest (RF) | {'max_depth': 10, 'max_features': 'sqrt', 'n_estimators': 10} |
|  | eXtreme Gradient Boosting (XGB) | {'learning_rate': 0.1, 'max_depth': 3, 'n_estimators': 1000, 'subsamples': 0.5} |
|  | Multi-Layer Perceptron (MLP) | {'activation': 'tanh', 'alpha': 0.0001, 'hidden_layer_sizes': (10, 20, 30), 'learning_rate': 'adaptive', 'solver': 'adam'} |
|  | Support Vector Machine (SVM) | {'C': 10, 'gamma': 0.0001, 'kernel': 'linear'} |
|  | Gradient Boosting (GB) | {'learning_rate': 0.1, 'max_depth': 7, 'n_estimators': 1000, 'subsample': 0.5} |
| Combined | Logistic Regression (LR) | {'C': 100, 'penalty': 'none', 'solver': 'newton-cg'} |
|  | Decision Tree (DT) | {'criterion': 'gini', 'max_depth': 11, 'max_features': None, 'max_leaf_nodes': 50, 'min_samples_leaf': 2, 'min_weight_fraction_leaf': 0.1, 'splitter': 'random'} |
|  | Random Forest (RF) | {'max_depth': 5, 'max_features': 'sqrt', 'n_estimators': 1000} |
|  | eXtreme Gradient Boosting (XGB) | {'learning_rate': 0.01, 'max_depth': 7, 'n_estimators': 1000, 'subsamples': 0.5} |
|  | Multi-Layer Perceptron (MLP) | {'activation': 'tanh', 'alpha': 0.0001, 'hidden_layer_sizes': (10, 20, 30), 'learning_rate': 'constant', 'solver': 'adam'} |
|  | Support Vector Machine (SVM) | {'C': 1, 'gamma': 0.0001, 'kernel': 'linear'} |
|  | Gradient Boosting (GB) | {'learning_rate': 0.001, 'max_depth': 3, 'n_estimators': 1000, 'subsample': 0.5} |
